# Institutional patterns in diagnosis and antifibrotic-era treatment uptake for IPF and PPF across U.S. centers

**DOI:** 10.1371/journal.pone.0360987

**Published:** 2026-10-22

**Authors:** Bradley A. Sheffield, Brian J. Cassel, Apostolos Perelas, Patricia Sime, Peter Jackson

**Affiliations:** 1 Virginia Commonwealth University School of Medicine, Richmond, Virginia, United States of America; 2 Division of Hematology, Oncology and Palliative Care, Virginia Commonwealth University, Richmond, Virginia, United States of America; 3 Division of Pulmonary and Critical Care Medicine, Virginia Commonwealth University, Richmond, Virginia, United States of America; SMS Medical College and Hospital, INDIA

## Abstract

**Purpose:**

For ten years, nintedanib and pirfenidone have been the only approved therapies for idiopathic pulmonary fibrosis (IPF), with nintedanib subsequently also approved for progressive pulmonary fibrosis (PPF). Previous studies demonstrate low antifibrotic (AF) uptake, but differences by site-of-care and AF uptake after seminal trials (IPF: INPULSIS/ASCEND; PPF: INBUILD) are not well characterized. We evaluated temporal trends in IPF and PPF diagnosis and AF use at academic (AC) versus non-academic (NonAC) centers.

**Methods:**

Using the U.S. TriNetX Collaborative Network, we compared IPF and PPF incidence, prevalence, and AF use by center type using linear regression across post-trial and post–FDA-approval windows (IPF: 2015–2017, 2015–2024; PPF: 2020–2022, 2020–2024). Slope differences (Δslope) were calculated as AC minus NonAC annual slopes; therefore, positive values indicate faster increases at ACs, whereas negative values indicate faster increases at NonACs.

**Results:**

Across both periods, IPF prevalence increased more rapidly at NonACs, while AF use was higher at ACs (2024: 33.5% vs 20.1%, *p* < 0.001). During the post-approval period (2015–2024), AF initiation and overall use rose faster at ACs: (AC–NonAC Δ slope +0.26 percentage points/year, p = 0.002; + 1.25 percentage points/year, p < 0.001). During the post-INPULSIS/ASCEND period (2015–2017), overall AF use rose faster at ACs (Δ slope +1.23 percentage points/year, p = 0.009), while AF initiation slopes did not differ significantly (Δ slope −0.22 percentage points/year, **p = 0.155**). PPF prevalence was similar across centers, with higher AF use at ACs in 2024 (17.1% vs 9.8%, *p* < 0.001). During the post-approval period (2020–2024), AF initiation and overall AF use increased faster at ACs (Δslope +1.16 percentage points/year, *p* < 0.001; + 1.94 percentage points/year, *p* = 0.009). During the post-INBUILD period (2020–2022), AF initiation rose faster at ACs (Δ slope +1.42 percentage points/year, p = 0.002), while overall AF slope differences were not significant. Outcome patterns for IPF revealed higher mortality and lung transplant in ACs, but lower respiratory failure and oxygen use. PPF showed higher respiratory failure and mortality in ACs, and no significant differences in lung transplant or oxygen use.

**Conclusions:**

AF use in IPF and PPF was higher and rose faster at ACs, suggesting faster AC adoption and potential barriers at NonACs. Mixed outcome findings suggest referral and severity bias may partially explain differences, however, are unlikely to fully account for them. More research is needed to account for referral and severity bias. Given the small NonAC PPF cohort (n = 216), PPF findings should be interpreted with caution.

## Introduction

Idiopathic pulmonary fibrosis (IPF) is the most well-characterized progressive fibrotic interstitial lung disease (ILD) marked by fibroblast proliferation and permanent scarring [1]. Progressive pulmonary fibrosis (PPF) encompasses heterogeneous non-IPF ILDs, including systemic autoimmune rheumatic disease–associated ILD (SARD-ILD), chronic hypersensitivity pneumonitis, and others, with a phenotype of progressive fibrosis and functional decline [2]. Both IPF and PPF are incurable chronic, life-limiting conditions and carry significant mortality [1,3]. Antifibrotics (AF) marked a paradigm shift as the first treatments to slow lung function decline in fibrotic lung disease [4–6].

The 2014 INPULSIS and ASCEND trials showed that nintedanib and pirfenidone, respectively, slowed FVC decline in IPF, leading to FDA approval [4,5]. INBUILD (2019) demonstrated similar benefits for nintedanib in PPF, resulting in FDA approval in 2020 [6]. These drugs remained the only approved therapies until nerandomilast was approved for IPF in October, 2025 [7,8].

Real-world AF uptake in IPF varies, from only ~25% in administrative claims to ~70% in registry cohorts [9–11]. In PPF, nintedanib use ranges from ~20−46% across U.S. and European cohorts [12–14]. Prior work has revealed treatment disparities in IPF among elderly, undernourished, Black and female patients [9–11,15]. PPF diagnosis is complex, requiring an underlying ILD with evidence of progression. A dedicated International Classification of Diseases, Tenth Revision (ICD-10) code (J84.170) was introduced in 2020 to support diagnosis and treatment [2,16,17]. Because PPF diagnosis relies on multidisciplinary ILD expertise which may be more available at ACs [2,18,19], ACs may be better positioned to support diagnosis and treatment. AF therapy is also costly and carries side effects that may require ongoing management [4–6,9,10], which may be more challenging in centers without dedicated ILD infrastructures [18,19]. Together, these factors may disproportionately hinder care at NonACs, however center-level differences have not previously been investigated and are poorly understood.

To address these gaps, we leveraged the TriNetX Collaborative Network to compare IPF (2015–2024) and PPF (2020–2024) incidence, prevalence, and AF uptake across ACs and NonACs. We also assessed demographic predictors of treatment and clinical outcomes across settings. To our knowledge, this is the first large-scale, longitudinal analysis using a U.S. EHR network to compare the effect of site-of-care, AC versus NonAC, on patterns of diagnosis and AF uptake in IPF and PPF.

## Methods

### Data source and study design

We conducted a multicenter retrospective cohort study using the TriNetX U.S. Collaborative Network, a de-identified, federated, HIPAA-compliant EHR dataset, derived from participating U.S. healthcare organizations. Institution-level identifiers other than center type (academic, non-academic, unknown), as well as geographic locations of participating healthcare organizations, were not available for analysis. All data was queried from April 1 to August 1, 2025. Full list of ICD-10 codes used can be found in Table 1 of Supplement 1.

**Table 1:** IPF regression summary. Δ slope was calculated as the annual slope at academic centers minus the annual slope at non-academic centers. Negative values indicate faster increases at non-academic centers, whereas positive values indicate faster increases at academic centers. AC = academic center; NonAC = non-academic center; AF = antifibrotic, pp/yr = percentage points per year.

IPF Diagnosis and Treatment Regression Summary					
Domain	Era / analysis period	AC slope	NonAC slope	AC − NonAC	p-value
Incidence, per 100k/yr	Post-approval, 2015–2024	0.52	1.34	−0.82	0.009
Prevalence, per 100k/yr	Post-approval, 2015–2024	5.02	9.36	−4.34	<0.001
AF initiation, pp/yr	Post-approval, 2015–2024	0.749	0.486	+0.263	0.002
Overall AF use, pp/yr	Post-approval, 2015–2024	3.285	2.033	+1.252	<0.001
Incidence, per 100k/yr	Post INPULSIS/ASCEND	0.00	3.00	−3.00	Not estimable
Prevalence, per 100k/yr	Post INPULSIS/ASCEND	4.50	8.00	−3.50	0.007
AF initiation, pp/yr	Post INPULSIS/ASCEND	0.329	0.552	−0.223	**0.155**
Overall AF use, pp/yr	Post INPULSIS/ASCEND	2.534	1.309	+1.225	0.009

### Ethics statement

All analyses were conducted entirely within the TriNetX platform using de-identified data. Under Virginia Commonwealth University’s approved *Research Using TriNetX* Standard Operating Procedure, research conducted entirely within the TriNetX platform does not require regulatory approval. Accordingly, IRB review and informed consent were not required.

### Study population

The IPF cohort included adults ≥18 years with ≥1 ICD-10 code J84.112 (2010–2024) without J84.170. The PPF cohort included adults ≥18 years with ≥1 ICD-10 code J84.170 (2018–2024) without J84.112. Trend regressions were restricted to post-trial and post–FDA-approval periods (IPF: 2015–2017, 2015–2024; PPF: 2020–2022, 2020–2024). The PPF ICD-10 code (J84.170) was introduced in 2020; pre-2020 PPF and pre-approval IPF values were included to demonstrate baseline and minimal off-label use but were not included in center-type analyses. Patients were categorized by care setting (AC, NonAC, or unclassified), predefined by TriNetX. “All centers” combined AC, NonAC, and unclassified. Center-type comparisons were limited to AC versus NonAC and excluded unclassified centers.

### Therapy

AF use was defined as ≥1 nintedanib or pirfenidone prescription for IPF, and nintedanib alone for PPF. For each calendar year, AF initiation was calculated as the percentage of patients with a first recorded AF prescription, and AF use was calculated as the percentage of patients with at least one AF prescription recorded during that year.

### Variables and outcomes

Demographic variables previously shown to be associated with AF treatment including sex (dichotomized), race (Black vs White, with other races excluded given small numbers of patients), as well as BMI (<18.5 vs ≥ 18.5 kg/m^2^, underweight vs non-underweight per WHO/CDC) and age at diagnosis were analyzed. Outcomes including all-cause mortality, oxygen use, respiratory failure, and lung transplantation by center type were compared to evaluate whether disease severity may contribute to observed differences in AF uptake. Full list of codes can be found in Supplement 1, Table 1.

### Statistical analysis

Annual incidence and prevalence per 100,000 were calculated in TriNetX. Linear regression with a center-type×year interaction in SPSS measured diagnosis and treatment trends across key time periods: (IPF: 2015–2017 [Post-INPULSIS/ASCEND], 2015–2024 [Post-FDA-Approval]; PPF: 2020–2022 [Post-INBUILD], 2020–2024 [Post-FDA-Approval]). Annual slope differences (Δslope) were defined as follows: NonACs were treated as the reference group and ACs as the comparison group. The interaction term therefore represents the AC minus NonAC annual slope difference. Positive slope differences indicate faster annual increases at ACs, whereas negative slope differences indicate faster increases at NonACs. Incidence and prevalence slope differences are reported as cases per 100,000 persons per year; AF initiation and treatment-use slopes are reported as percentage points per year (pp/yr). Differences in incidence, prevalence, and AF use by center type were assessed using Pearson χ^2^ tests. Propensity-matched logistic regression and Cox models in TriNetX evaluated demographic predictors of treatment, time-to-treatment after initial diagnosis, and clinical outcomes.

## Results

### Cohorts

A total of 59,890 adults with IPF (42,536 AC, 16,470 NonAC, and 884 unclassified), and 2,358 PPF patients (1,716 AC; 216 NonAC; 426 unclassified) were identified.

Cohort demographic distributions can be found in Supplement 1, Tables 2-4. Incidence, prevalence, and AF uptake data by center-type can be found in Supplement 2.

### IPF incidence and prevalence (Fig 1, Table 1)

Across all centers (2015–2024), IPF incidence increased from 11.0 to 19.0 per 100,000/year and prevalence from 34.0 to 94.0 per 100,000. IPF burden at ACs was higher initially, but by 2024 NonACs exceeded ACs in prevalence (109.0 vs 89.0 per 100,000, p < 0.001) and incidence (21.0 vs 18.0 per 100,000/year, p < 0.001). Incidence and prevalence rose faster at NonACs than ACs (1.34 vs 0.52 per 100,000 persons/year; Δ slope = −0.82; p = 0.009) and (9.36 vs 5.02 per 100,000 persons/year; Δ slope = −4.34; p < 0.001) respectively.

**Fig 1:**
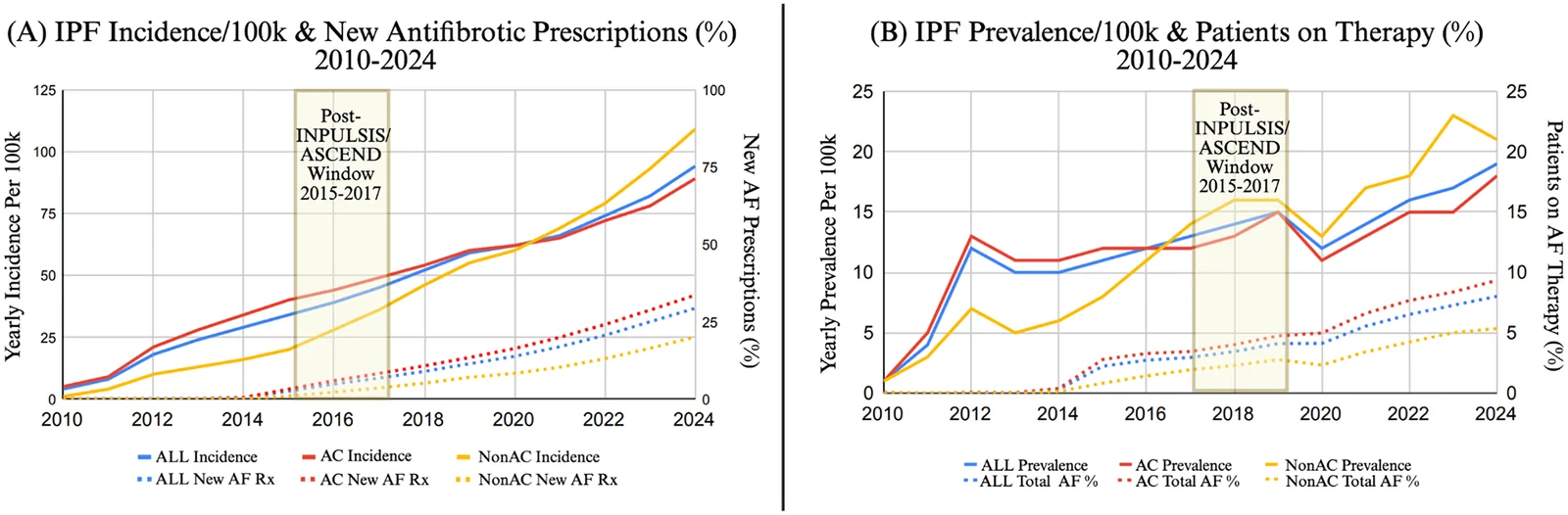
IPF incidence and new AF prescriptions (A) and IPF prevalence and percentage of patients on AF therapy (B), 2010–2024, by center type. Solid lines (left y-axis) show IPF incidence and prevalence per 100,000 population. Dotted lines (right y-axis) show (A) new AF prescriptions and (B) percentage of patients on therapy. Statistical analyses were based on post-INPULSIS/ASCEND (2015-2017) and post FDA approval (2015-2024) time periods; data preceding 2015 is shown to demonstrate baseline. Abbreviations; Idiopathic Pulmonary Fibrosis (IPF), Antifibrotic (AF), prescription (Rx).

Post-INPULSIS (2015–2017), incidence and prevalence rose faster at NonACs (~3.0 vs ~ 0.0 per 100,000 persons/year; p not estimable) and (8.0 vs 4.5 per 100,000 persons/year; Δ slope = −3.5; p = 0.007) respectively.

### IPF antifibrotic treatment trends (Fig 1, Table 1)

Across all centers (2015–2024), new AF prescriptions increased from 2.24% to 8.02% and percent of patients on AF therapy increased from 2.56% to 29.32%. By 2024, new AF prescriptions and percent of patients on therapy was higher at ACs than NonACs (9.3% vs 5.4%,*p* < 0.001; 33.53% vs 20.08%, *p* < 0.001). Over 2015–2024, new AF prescriptions were greater at ACs (0.749 pp/year vs 0.486 pp/year; Δslope = +0.263 pp/year; p = 0.002), similarly percent of patients on therapy was higher at ACs (3.285 vs 2.033 pp/year; Δ slope = +1.252 pp/year; p < 0.001).

Post-INPULSIS (2015–2017), new AF prescriptions were similar (0.329 pp/year vs 0.552 pp/year; Δ slope= −0.223 pp/year; **p = 0.155)**, while percent of patients on AF therapy rose faster at ACs (2.534 pp/yr vs 1.309 pp/yr; Δ slope = +1.225 pp/year; p = 0.009). All-time odds of AF receipt favored ACs (OR 1.71, 95% CI 1.59–1.83), and time-to-treatment was faster at ACs (HR 1.47, 95% CI 1.41–1.54).

### PPF incidence and prevalence (Fig 2, Table 2)

Across all centers (2020–2024), PPF incidence increased from 0.0 to 4.0 per 100,000/year and prevalence from 0.0 to 9.0 per 100,000. By 2024, incidence was higher at ACs than NonACs (5.0 vs 3.0 per 100,000/year, p < 0.001) while prevalence was similar (9.0 per 100,000 at both, **p = 1.000)**. Slopes did not differ by center type for incidence (1.20 vs 0.90 per 100,000 persons/year; slope Δ = +0.300; **p = 0.414)** or prevalence (2.20 vs 2.30 per 100,000 persons/year; slope Δ= −0.100; **p = 0.839).**

**Table 2:** PPF regression summary. Δ slope was calculated as the annual slope at academic centers minus the annual slope at non-academic centers. Negative values indicate faster increases at non-academic centers, whereas positive values indicate faster increases at academic centers. AC = academic center; NonAC = non-academic center; AF = antifibrotic, pp/yr = percentage points per year.

PPF Diagnosis and Treatment Regression Summary					
Domain	Era / analysis period	AC slope	NonAC slope	AC − NonAC	p-value
Incidence, per 100k/yr	Post-approval, 2020–2024	1.20	0.90	+0.300	**0.414**
Prevalence, per 100k/yr	Post-approval, 2020–2024	2.20	2.30	−0.100	**0.839**
AF initiation, pp/yr	Post-approval, 2020–2024	1.58	0.42	+ 1.16	<0.001
Overall AF use, pp/yr	Post-approval, 2020–2024	3.98	2.03	+1.94	0.009
Incidence, per 100k/yr	Post-INBUILD (2020–2022)	0.50	1.00	−0.500	**0.225**
Prevalence, per 100k/yr	Post-INBUILD (2020–2022)	1.00	1.50	−0.500	**0.225**
AF initiation, pp/yr	Post-INBUILD (2020–2022)	1.454	0.034	+1.420	0.002
Overall AF use, pp/yr	Post-INBUILD (2020–2022)	2.818	1.099	+1.719	**0.080**

**Fig 2:**
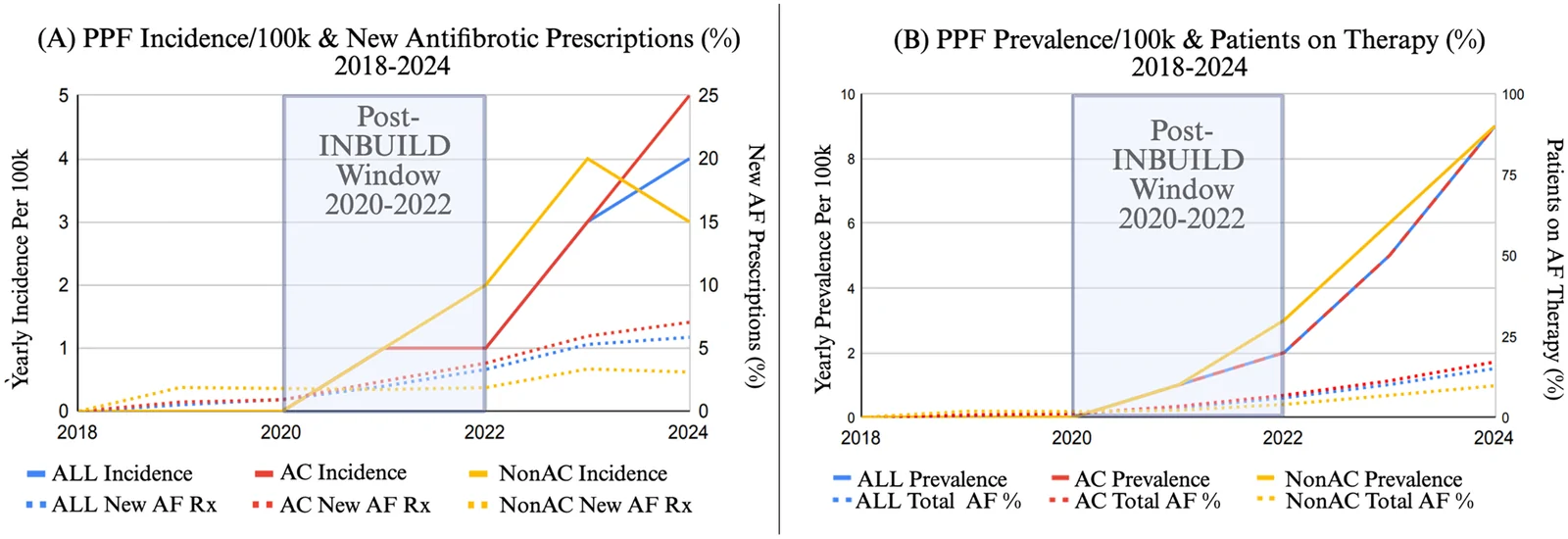
PPF incidence and new AF prescriptions (A) and PPF prevalence and percentage of patients on AF therapy (B), 2018–2024, by center type. Solid lines (left y-axis) show PPF incidence and prevalence per 100,000 population. Dotted lines (right y-axis) show (A) new AF prescriptions and (B) percentage of patients on therapy. Statistical analyses were based on post-INBUILD (2020-2022) and post FDA approval (2020-2024) time periods; data preceding 2020 is shown to demonstrate baseline. Abbreviations; Progressive Fibrosing Interstitial Lung Disease (PPF), Antifibrotic (AF), prescription (Rx).

Post-INBUILD (2020–2022) incidence and prevalence trended higher at NonACs, though not significant (slope Δ= −0.500 per 100,000 persons/year for both; **p = 0.225)**

### PPF antifibrotic treatment trends (Fig 2, Table 2)

Across all centers (2020–2024), new AF prescriptions reached 5.86% and percent of patients on AF therapy reached 15.10%. By 2024, new AF prescriptions and percent of patients on therapy were higher at ACs than NonACs (7.05% vs 3.11%, *p* = 0.001; 17.13% vs 9.77%, *p* < 0.001). From 2020–2024, new AF prescriptions increased faster at ACs (+1.58 pp/year vs + 0.42 pp/year; slope Δ = + 1.16 pp/year; p < 0.001) and percent of patients on therapy rose faster at ACs (+3.98 pp/year vs + 2.03 pp/year; slope Δ= + 1.94 pp/year; p = 0.009).

Post-INBUILD (2020–2022), new AF prescriptions rose faster at ACs (1.454 pp/yr vs 0.034 pp/yr; slope Δ = +1.420 pp/year; p = 0.002). Percent of patients on therapy was similar (2.818 pp/yr vs 1.099 pp/yr; slope Δ = +1.719 pp/year; **p = 0.080).** All-time center-type odds and time-to-treatment in PPF were similar (OR 1.32, 95% CI 0.63–2.75; HR 1.14, 95% CI 0.83–1.55).

AF adoption overview across combined IPF and PPF cohorts (Fig 3)

**Fig 3:**
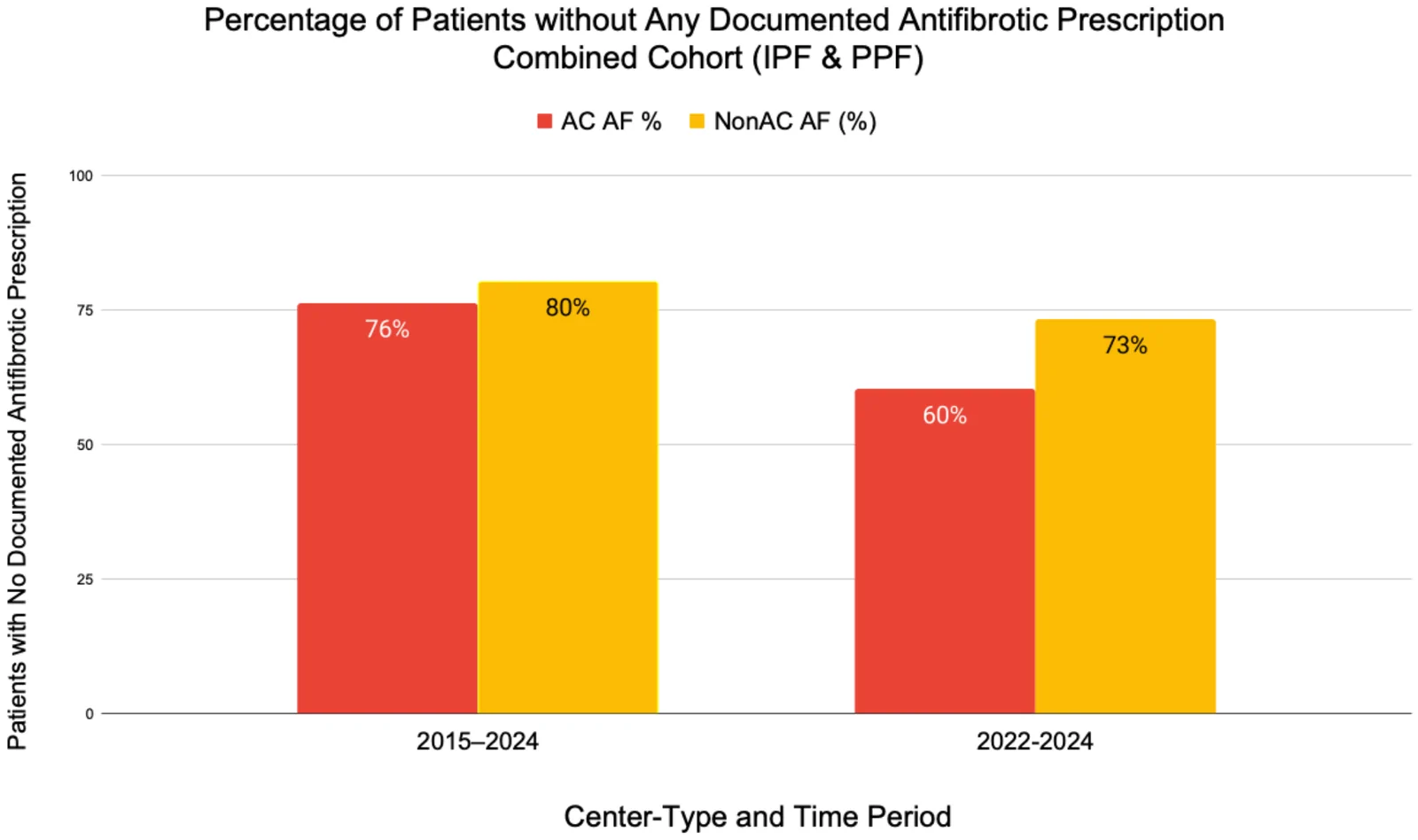
Antifibrotic prescribing in a combined cohort of guideline-eligible IPF and PPF patients. Antifibrotic prescribing was defined as ≥1 recorded prescription during the specified period. Across both AC and NonAC, most patients had no recorded antifibrotic prescription in 2015–2024, with persistent gaps despite increased use in 2022–2024.

To summarize AF use across diseases with guideline-recommended therapy, we evaluated prescribing in a combined IPF and PPF cohort. This secondary analysis was conducted separately at a different time point; consequently, its denominators are not directly comparable to the disease-specific cohort totals reported above. From 2015–2024, most patients had no recorded AF prescription (AC: 76% [25,350/33,059]; NonAC: 80% [11,114/13,941]; p < 0.001). This gap persisted in 2022–2024 (AC: 60% [10,429/17,502]; NonAC: 73% [5,335/7,319]; p < 0.001

### Patient-level treatment predictors (Fig 4)

In IPF, treatment was more common in White patients (OR 1.60, 95% CI 1.43–1.79), males (OR 1.49, 95% CI 1.42–1.56), and those with BMI > 18.5 (OR 1.78, 95% CI 1.40–2.27). These patterns were broadly similar by center type, but the race effect was strongest at ACs (OR 1.78, 95% CI 1.55–2.05) and was not seen at NonACs (OR 1.00, 95% CI 0.82–1.23). In Cox models, males had a higher hazard of treatment initiation than females (HR 1.58, 95% CI 1.52–1.64). White race also had a higher hazard of treatment initiation than Black patients (HR 1.67, 95% CI 1.54–1.82) (Fig 4).

**Fig 4:**
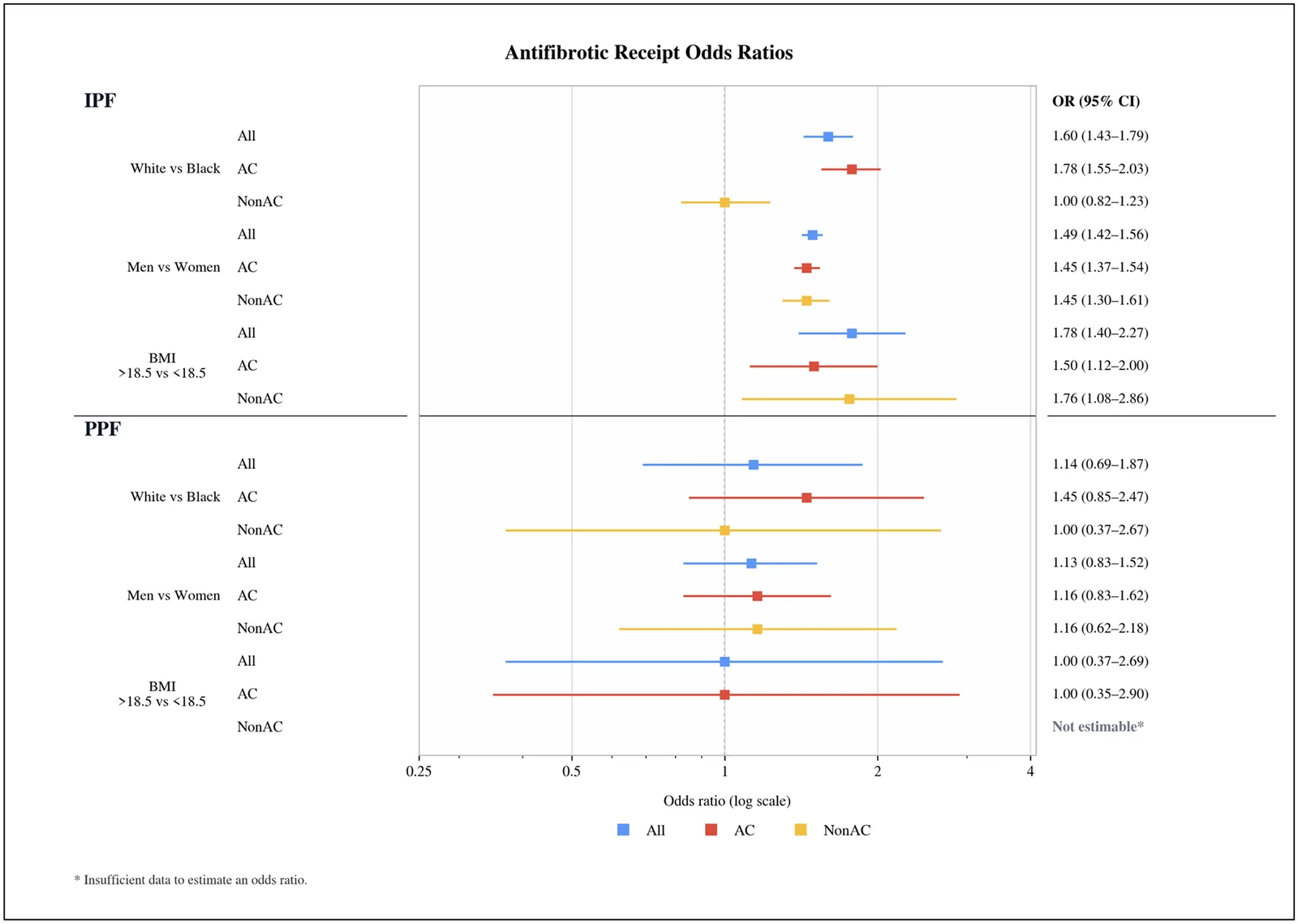
IPF and PPF odds of AF treatment by dichotomized demographic factors, stratified by center type. Among propensity score–matched patients, bivariate logistic regressions assessed whether sex (male vs female), race (Black vs White), or BMI (>18.5 vs ≤ 18.5) were associated with AF treatment.

In PPF, no treatment predictors reached statistical significance.

### Outcomes by center type

Severity data was limited by lack of PFT data. To investigate differences in severity of illness by center-type, clinical outcomes were investigated. In IPF, AC care was associated with higher mortality (HR 1.21, 95% CI 1.16–1.26) and lung transplant (HR 1.12, 95% CI 1.03–1.22), but lower hazards of respiratory failure (HR 0.82, 95% CI 0.78–0.86) and oxygen use (HR 0.82, 95% CI 0.78–0.86).

In PPF, respiratory failure (HR 1.87, 95% CI 1.22–2.85) and death (HR 2.76, 95% CI 1.66–4.60) were higher at ACs, with a nonsignificant trend toward increased oxygen use (HR 1.51, 95% CI 0.99–2.31). Lung transplant was rare and similar between center types (HR 0.76, 95% CI 0.23–2.67).

## Discussion

In this large multicenter retrospective analysis, we examined IPF (2015–2024) and PPF (2020–2024) incidence, prevalence, and AF use across ACs and NonACs. We assessed uptake by examining periods after seminal trials, identified patient-level treatment predictors, and explored outcomes by center-type.

Overall, IPF prevalence increased across all sites, reaching 89 per 100,000 at ACs and 109 per 100,000 at NonACs in 2024, higher than prior population-based estimates but lower than U.S. Veterans Affairs cohorts [20–24]. Rising prevalence likely reflects increased awareness, more consistent coding, landmark AF trials, and earlier detection, rather than necessarily reflecting a true increase in underlying disease frequency. Improved survival in the AF era may also contribute modestly, though survival data remain inconclusive and any benefit appears modest at best. Increased incidence may reflect improved recognition following the 2018 ATS/ERS/JRS/ALAT guideline update and heightened awareness as above with AF availability [25]. In addition, we observed a 2020 dip followed by a 2021–2023 rebound likely related to a COVID-related disruption in ILD care and evaluation with a post-pandemic diagnostic “catch-up” period.

By 2024, PPF prevalence was 9 per 100,000 at both center types, lower than many prior estimates [16,17,24,26], which is expected given that earlier studies relied on sensitive proxy definitions whereas we used a dedicated, specific PPF code. Similar to IPF, we attribute the dramatic rise in cases to increased recognition and coding practices rather than a reflection of true epidemiology. Although this approach likely underestimates true disease burden, ICD-10 coding reflects clinician recognition and is often required for insurance coverage of AF [26–29], providing a pragmatic lens on treatment patterns. Despite anticipating higher diagnostic rates at ACs due to consistent multidisciplinary expertise and referral patterns [18,19], incidence was similar across center types and IPF prevalence was higher at NonACs, potentially reflecting differences in disease burden, recognition, or coding practices.

AF uptake rose more rapidly at ACs, resulting in apparent treatment gaps of 13.4% for IPF and 7.3% for PPF by 2024, suggesting faster translation of evidence into care at ACs. Although statistically significant differences were observed in PPF AF uptake, these findings should be interpreted with caution given the small NonAC sample size (n = 216). Lower uptake at NonACs may reflect differences in expertise, diagnostic certainty, and prescribing workflows, as well as potential residual confounders. Because disease severity could not be fully captured, these observed treatment differences may be partly subject to confounding by severity at ACs. Outcome patterns partially support this concern: mortality and lung transplantation were higher at ACs, suggesting greater disease severity among patients treated at ACs; however, respiratory failure and oxygen use were lower at ACs, suggesting the opposite. Overall, outcomes were mixed and insufficient to support or exclude confounding by severity. Disease severity differences may contribute to observed AF differences by center type, but they do not appear to fully explain them.

Differences in AF uptake may also be partly explained by unmeasured referral bias, whereby patients diagnosed at NonACs are referred to ACs for further management, including treatment initiation. Such referral patterns may themselves represent a geographic access barrier, as patients living farther from ACs may face delayed specialty evaluation and treatment initiation. In ILD, living more than 70 km from a specialty clinic is associated with faster progression to death or lung transplant [30]. These barriers may contribute to inequities in in ILD care and underscore the need to further examine potential obstacles to AF use at NonACs.

Despite apparent higher AF use at ACs, treatment remained suboptimal, with many potentially eligible patients never receiving therapy. In a combined IPF and PPF analysis, most patients had no recorded AF prescription even from 2022–2024 (ACs 60% vs NonACs 73%). Miscoding, modest efficacy and side effects may contribute to this low uptake: discontinuation rates range from 14% to 66% across trial and real-world settings [4–6,9–12,15]. However, many patients tolerate therapy, and discontinuation is often temporary and managed with symptom control, dose reduction, and later resumption [4–6,]. Given the magnitude of untreated IPF and PPF patients it is reasonable to assume that a proportion of patients never prescribed AFs may benefit from a trial of therapy.

Patient-level AF treatment patterns in IPF were similar to prior work [9,10], with higher use and faster time-to-treatment among white patients and men. Notably, race-based differences were not observed at NonACs. Because white men comprise most IPF cases [1,18–20], this may partly reflect greater diagnostic certainty. Guideline reliance on radiographic features more commonly observed in men may also contribute [1,21,25]. Non-underweight patients also had higher odds of AF receipt, likely reflecting concerns about initiating and managing therapy in underweight patients given the gastrointestinal side-effect profile; this finding highlights the need to better understand how certain patient characteristics influence treatment decision-making and supportive care in this population. In PPF, we did not detect sex, racial, and BMI-based disparities, though the analysis may have been underpowered.

## Conclusion

We identified institutional and patient-level differences in AF treatment for IPF and PPF. Despite higher IPF burden at NonACs and similar PPF burden across center types, ACs appeared to adopt AFs more rapidly, suggesting persistent treatment gaps. While outcome data suggests possible referral and severity bias, they are mixed and are unlikely to fully explain center-type differences. Additionally, PPF findings should be interpreted cautiously given the small NonAC sample size. White race was associated with AF treatment in IPF at ACs but not at NonACs, while male sex and BMI ≥ 18.5 were also associated with treatment. Our findings underscore the need for further study to identify barriers to guideline-based ILD treatment and for prospective efforts to improve equitable access to treatment. Future center-type analyses with more detailed referral and disease-severity data are needed to distinguish true institutional differences from potential confounding.

## Limitations

This study has important constraints. TriNetX captures prescriptions recorded within participating systems, so prescriptions outside the network and nonadherence may not be captured; absence of an EHR prescription does not necessarily indicate no therapy. We could not fully account for referral patterns or cross-facility co-management, including patients diagnosed at one site and treated at another. TriNetX AC/NonAC classification is operational and likely heterogeneous within each group. As a federated network, TriNetX is subject to data refreshes and changes in contributing sites. These features, together with a small NonAC PPF sample and uncertain representation of community and private-practice settings, limit inferences about center-level effects. Mortality capture and coding of respiratory failure and oxygen use may vary across healthcare organizations. Therefore, apparent differences in these outcomes by center type may reflect differential outcome ascertainment as well as differences in case mix or disease severity. Reliance on ICD-10 coding is vulnerable to undercoding and misclassification, and adjudication of patient level data is not possible within the database. Post-trial trend analyses were based on regression models using only three annual data points; consequently, one comparison was not estimable, and the associated P values should be interpreted cautiously. Finally, limited PFT availability constrained adjustment for disease severity.

## Supporting information

S1 File Definitions used in TriNetX. Definitions and coding criteria used to identify study populations and outcomes.
(DOCX)

S2 Dataset Center-type dataset. Supplementary dataset containing associated study data.
(XLSX)
