# Supplementary material for: Institutional patterns in diagnosis and antifibrotic-era treatment uptake for IPF and PPF across U.S. centers
Source: PLoS One. 2026 Oct 22;21(10):e0360987. doi: 10.1371/journal.pone.0360987 (PMC13690216; doi:10.1371/journal.pone.0360987)
Supplement: Supplementary file 1 [file pone.0360987.s001.docx]

**Table S1. Variable Definitions for cohorts & outcomes**

| Variable | Code system / TriNetX concept | Code / concept |
| --- | --- | --- |
| IPF | ICD-10-CM | J84.112 |
| PPF | ICD-10-CM | J84.170 |
| Nintedanib | RxNorm | 1592737 |
| Pirfenidone | RxNorm | 1592254 |
| Respiratory failure | ICD-10-CM | J96.0; J96.00, R06.03, J96.02 |
| All-cause mortality | Outcome variable | Deceased |
| Lung transplant | ICD-10-CM / CPT / SNOMED CT | Z94.2; 32851; 232658009; 33.5 |
| Oxygen use | ICD-10-CM / RxNorm | Z99.81; 7806 |

**Table S2. Source Population Demographics by Center Type**

| Characteristic | Overall source population | Academic source population | Non-academic source population |
| --- | --- | --- | --- |
| Total patients, N | 37,589,544 | 26,668,051 | 10,915,233 |
| Age, mean ± SD | 52 ± 21 | 52 ± 21 | 51 ± 21 |
| Age range, years | 18–90 | 18–90 | 18–90 |
| Female, % | 55.78 | 55.92 | 55.43 |
| Male, % | 44.17 | 44.04 | 44.49 |
| Unknown sex, % | 0.05 | 0.04 | 0.08 |
| White, % | 70.22 | 70.80 | 68.81 |
| Black or African American, % | 11.48 | 11.80 | 10.69 |
| Asian, % | 3.78 | 2.49 | 6.95 |
| Other race, % | 4.07 | 4.42 | 3.21 |
| American Indian or Alaska Native, % | 0.51 | 0.56 | 0.38 |
| Native Hawaiian or Other Pacific Islander, % | 0.61 | 0.18 | 1.66 |
| Unknown race, % | 9.33 | 9.75 | 8.30 |
| Not Hispanic or Latino, % | 67.19 | 67.01 | 67.62 |
| Hispanic or Latino, % | 9.36 | 9.84 | 8.21 |
| Unknown ethnicity, % | 23.45 | 23.15 | 24.17 |

**Table S3. IPF Population Demographics by Center Type**

| Characteristic | All IPF (N=59,890) | AC IPF (N=42,536) | NonAC IPF (N=16,470) |
| --- | --- | --- | --- |
| Age, mean ± SD | 76 ± 11 | 77 ± 11 | 78 ± 10 |
| Age range, years | 18–90 | 18–90 | 18–90 |
| Male, % | 58.33 | 58.43 | 56.70 |
| Female, % | 41.64 | 41.54 | 43.28 |
| Unknown sex, % | 0.03 | 0.03 | 0.08 |
| White, % | 76.33 | 76.49 | 80.92 |
| Black or African American, % | 8.67 | 8.55 | 6.66 |
| Asian, % | 2.52 | 2.49 | 6.29 |
| Other race, % | 3.44 | 3.42 | 1.50 |
| American Indian or Alaska Native, % | 0.77 | 0.76 | 0.29 |
| Native Hawaiian or Other Pacific Islander, % | 0.07 | 0.07 | 0.78 |
| Unknown race, % | 8.20 | 8.22 | 3.56 |
| Not Hispanic or Latino, % | 73.10 | 72.96 | 72.04 |
| Hispanic or Latino, % | 7.03 | 7.00 | 6.92 |
| Unknown ethnicity, % | 19.87 | 20.04 | 21.04 |

**Table S4. PPF Population Demographics by Center Type**

| Characteristic | All PPF (N=2,358) | AC PPF (N=1,716) | NonAC PPF (N=216) |
| --- | --- | --- | --- |
| Age, mean ± SD | 71 ± 13 | 70 ± 13 | 72 ± 12 |
| Age range, years | 18–90 | 20–90 | 18–90 |
| Female, % | 57.04 | 57.72 | 54.63 |
| Male, % | 42.96 | 42.28 | 45.37 |
| Unknown sex, % | 0.00 | 0.00 | 0.00 |
| White, % | 68.36 | 66.86 | 73.14 |
| Black or African American, % | 17.30 | 18.57 | 13.27 |
| Asian, % | 3.25 | 2.54 | 5.70 |
| Other race, % | 3.43 | 3.27 | 4.01 |
| American Indian or Alaska Native, % | 0.91 | 1.04 | 1.54 |
| Native Hawaiian or Other Pacific Islander, % | 0.35 | 0.45 | 1.54 |
| Unknown race, % | 6.58 | 7.64 | 2.93 |
| Not Hispanic or Latino, % | 78.66 | 79.70 | 75.00 |
| Hispanic or Latino, % | 6.31 | 6.51 | 5.56 |
| Unknown ethnicity, % | 15.03 | 13.79 | 19.44 |
